# Legal status disparities in preventive care usage among Latino immigrants in California: A cross-sectional analysis

**DOI:** 10.1371/journal.pgph.0004855

**Published:** 2025-07-01

**Authors:** Tianjian Lai, Brian Tuohy, Roger Waldinger

**Affiliations:** 1 Department of Medicine and Center for Health and Social Sciences, The University of Chicago, Chicago, Illinois, United States of America; 2 Lewis Katz School of Medicine, Temple University, Medical Education & Research Building, Philadelphia, Pennsylvania, United States of America; 3 Department of Sociology, UCLA, Los Angeles, California, United States of America; The Ohio State University, UNITED STATES OF AMERICA

## Abstract

This paper examines disparities in preventive care utilization among Latino immigrants, differentiating between naturalized U.S. citizens, permanent residents, temporary status holders, and undocumented immigrants. We analyzed data from the 2015–2016 California Health Interview Survey (CHIS), a cross-sectional, representative sample of California residents. Our sample consisted of 5,513 respondents who self-identified as foreign-born Latinos. We examined three measures of preventive health: annual checkups, flu vaccinations, and access to mammogram screenings. We applied weighted logistic regression to estimate how immigrants’ odds of receiving annual checkups and flu vaccinations in the past year, and mammogram screenings in the past two years, differed by legal status after controlling for various sociodemographic and health factors. We found that naturalized citizens and lawful permanent residents (LPRs) report higher unadjusted odds of having annual checkups (citizen OR = 3.23, p = 0.000; LPR OR = 2.03, p = 0.000), flu vaccines (citizen OR = 2.14, p = 0.000; LPR OR = 1.62, p = 0.000), and mammograms (citizen OR = 3.24, p = 0.000; LPR OR = 1.95, p = 0.005) relative to undocumented immigrants. However, immigrants with temporary legal status do not differ significantly in their rate of preventive care utilization relative to those who are undocumented. This suggests that precarious legal status, rather than outright undocumented status alone, contributes to disparities in preventive care. After controlling for sociodemographic, health status, and health access factors, legal status disparities in flu vaccinations lose statistical significance, while naturalized citizens continue to have higher odds of annual checkups (OR = 1.46, p = 0.032) and mammograms (OR = 2.69, p = 0.000) and permanent residents remain more likely to receive mammograms (OR = 1.68, p = 0.05) relative to undocumented immigrants. Our findings underscore the need to address access to and utilization of preventive care among legally vulnerable immigrants.

## 1. Introduction

Preventive healthcare plays a significant role in improving patients’ quality of life and reducing their risk of serious illness, disability, or death from treatable causes. Higher rates of preventive care in the United States could potentially save over 2 million life-years annually [1]. Furthermore, certain forms of preventive care, especially vaccinations, inhibit the spread of infectious diseases and are essential for public health. Nonetheless, preventive care is underutilized in the United States. Only 8 percent of U.S. adults aged 35 or older have received all the recommended high-priority preventive services [2]. In addition, access to preventive care is stratified within the U.S. population: Latino immigrants in particular report lower rates of preventive care access and vaccine coverage [3,4].

Previous studies have uncovered several factors that may constrain Latino immigrants’ access to preventive care. Latino immigrants have high rates of employment but often work in low-wage jobs without employer-based health insurance, leading to low rates of insurance coverage with correspondingly constrained access to preventive services [5,6]. Language barriers and residence in rural areas where medical care is limited may also prevent Latino immigrants from accessing comprehensive care or understanding available healthcare opportunities [7,8].

Less examined is the critical role of legal status. Among Latino immigrants in the United States, an estimated 43% are naturalized citizens, 31% are lawful permanent residents (LPRs) or visa holders, and 25% are undocumented [9]. Relative to naturalized citizens and LPRs, the roughly 11 million undocumented immigrants in the United States use fewer medical services and have lower healthcare expenditures [10,11]. Undocumented immigrants may be deterred from accessing medical services due to their low rates of insurance coverage, discomfort navigating U.S. healthcare, and fears of deportation [12–14]. Past studies have found that undocumented Mexican immigrants are less likely to report having a usual source of health care or to have seen a physician in the past year relative to documented Mexican immigrants, attributing this reduced access to care to factors such as lower rates of insurance coverage, shorter time in the United States, and lower rates of English proficiency [15]. Enforcement targeting undocumented immigrants also aggravates stressors that contribute to poor health and generates fears that deter the use of preventive services [16–18]. Furthermore, undocumented immigrants are generally ineligible for public insurance programs such as Medicaid and Medicare, with their direct exclusion from the Affordable Care Act (ACA) widening legal status disparities in insurance coverage [19]. While the Emergency Medical Treatment and Active Labor Act (EMTALA) requires hospitals to provide emergency treatment to all patients regardless of their legal status and ability to pay, EMTALA does not make allowances for non-emergency care such as preventive care.

Due in part to the sensitivity of asking immigrants about their legal status, data sources containing detailed information on both immigrants’ healthcare access and their legal status are scant. Consequently, few studies have empirically and quantitatively disaggregated legal status differences in immigrants’ access to preventive care. Using the 2007 Hispanic Health Care Survey, Rodriguez, Vargas Bustamante, and Ang (2009) find that relative to U.S.-born Latinos, undocumented Latino immigrants have lower likelihoods of receiving blood pressure and cholesterol checks [20]. Likewise, Pourat et al. (2014) analyze data from the 2009 California Health Interview Survey to find that undocumented immigrant women aged 50 and older were significantly less likely to have received a mammogram in the past 5 years relative to their U.S.-born peers [10]. Nonetheless, more work is needed to examine legal status disparities in Latino immigrants’ preventive care access. We know little about legal status trends in preventive care usage in the years following ACA implementation, which categorically excludes undocumented immigrants from expanded healthcare access. Similarly, disparities among additional categories of preventive care, including vaccinations, have yet to be studied. Furthermore, few studies have examined healthcare access among immigrants in the United States on temporary legal statuses, a group representing roughly 5% of the foreign-born population [21].

Our study begins to address these important gaps by analyzing disparities in preventive care utilization among Latino immigrants varying in legal status: naturalized U.S. citizens, LPRs, temporary status holders, and undocumented immigrants. We define temporary status holders as individuals who have documents that authorize their temporary stay in the United States, such as a visa or work permit. By distinguishing temporary status holders, who lack a pathway to permanent residence but who are authorized to reside in the United States on a time-limited basis, from undocumented immigrants, whose U.S. presence is unauthorized, we capture a broader range in rights and entitlements than prior research has been able to identify.

We focus our analyses on three critical types of preventive care for which we know little of how utilization rates differ by immigrants’ legal status. We consider annual checkups, where medical professionals screen patients for conditions such as hypertension, ensure that vaccines and medications are up-to-date, and discuss lifestyle changes such as smoking cessation. Health checks have been found to increase detection of chronic diseases, utilization of other preventive services such as cancer screenings, improve patient-reported health and quality of life outcomes, and foster close relationships between patients and doctors [22,23]. We also examine annual flu vaccines, which are estimated to save 24 life years per 10,000 people annually and are important for the wellbeing of not only vaccine recipients, but also the general population [1]. Latinos in the United States report lower flu vaccination rates relative to Whites, which may explain their higher age-adjusted rates of flu hospitalization and intensive care unit admission [24,25]. Finally, we consider immigrant women’s access to mammogram screenings. Breast cancer is the leading cause of cancer death among Latina women in the United States, who report lower rates of mammography utilization relative to non-Hispanic Black and white women [26,27]. Furthermore, undocumented Latina patients have been found to be diagnosed with breast cancer at an earlier age and more advanced stage compared to documented Latina patients and report longer time in between diagnoses of breast cancer and treatment [28]. Uncovering disparities in access to these important areas of preventive care will have significant implications across multiple health outcomes.

Our data centers on the case of California, home to the largest population of immigrants, including undocumented immigrants, in the United States [29]. California is a state with more favorable policies towards legally vulnerable immigrants, including permitting undocumented immigrants to obtain driver’s licenses, attend and receive in-state tuition and financial aid at public universities, and limiting employers’ use of E-Verify to examine employee’s work eligibility in the United States. Furthermore, as of 2024, California became the first state to expand Medicaid eligibility to undocumented residents. As such, any legal status disparities in access to preventive care found in California may be amplified in other states with less favorable policies towards immigrants with precarious legal status.

The implications of immigrants’ inadequate access to preventive care can extend to millions of individuals, encompassing not only the roughly 45 million immigrants living in the United States, but also their U.S.-born family and community members. Immigrants lacking preventive care may experience mortality at younger ages, prolonged suffering from debilitating illnesses, and lower quality of life. By leveraging a unique dataset, we provide evidence for critical disparities with significant individual- and population-level implications.

## 2. Materials and methods

### 2.1. Ethics statement

Data for this study comes from the California Health Interview Survey (CHIS) confidential data files, which contain variables measuring sensitive information such as respondents’ legal status. To ensure respondent confidentiality, we did not have access to confidential CHIS data directly. Instead, the data for this study was analyzed through the CHIS Data Access Center (DAC), which received approval to access this data from the UCLA South General Institutional Review Board (UCLA IRB #11–002227). Respondents to the 2015–2016 CHIS waves were recruited through random digit dialing of landlines and cell phones and gave their informed consent.

### 2.2. Study sample

We used data from the 2015–2016 waves of CHIS, a large-scale, population-representative survey of non-institutionalized residents in California, collected annually by the University of California Los Angeles Center for Health Policy Research. Due to its large-scale and population-representative sample and detailed information on respondents’ legal status and health outcomes, CHIS is a unique dataset well-suited for examining legal status differences in immigrants’ preventive health usage. The 2015 and 2016 waves of CHIS differentiate temporary status holders from undocumented immigrants and contains information on the utilization of key forms of preventive care such as annual checkups, flu vaccinations, and mammograms. More recent waves of CHIS either do not disaggregate the legal status of immigrants who are not permanent residents or lack the same breadth of preventive care measures. Although these data precede more recent Medicaid expansions in California and the post-2016 political climate, they capture a pivotal period following the initial rollout of the ACA and offer important historical insight.

We focused our analyses on Latino foreign-born respondents in the 2015 and 2016 waves of CHIS. Latinos make up the largest ethnic group in California, at 39% of the population, and are predominantly (83%) of Mexican ancestry [30]. Latinos in California are socioeconomically disadvantaged on several markers relative to other ethnic groups in the state. For instance, 17% of Latinos live below the federal poverty line, compared to 13% of Californians overall, and 35% did not complete high school, compared to 17% of Californians overall [30]. Latinos in California also report high levels of difficulty affording medical care. They are more likely to be uninsured (13% compared to 8% of Californians overall), with one in three noncitizen Latinos lacking insurance coverage [30]. Furthermore, 40% of Latinos report difficulty paying medical bills and 52% report having medical debt, more than any other ethnic group in California [31].

### 2.3. Measures

We examined immigrants’ access to three different types of preventive healthcare:

1. Annual checkups: a dichotomous variable, measuring whether the respondent went to a doctor or medical provider for a routine checkup in the last year;
2. Flu vaccinations: a dichotomous variable measuring whether the respondent received a flu vaccine in the past year;
3. Mammograms: a dichotomous variable measuring whether a female respondent over age 40 reported receiving a mammogram in the past 2 years.

Data on whether respondents received an annual checkup, and whether respondents received an annual flu vaccine are available for all adults ages 18 and up (N = 5,513). Data on mammogram access are available for women aged 40 and older (N = 2,292). Rates of utilization are stable between 2015 and 2016 for each of the preventive outcomes and legal status categories (see S1 Fig in supporting files).

Our main predictor of interest is immigrants’ legal status. We examined four legal status categories: naturalized citizen, LPR, temporary status holder (e.g., visa or work permit), and undocumented. Our analyses include several demographic and health-related controls: gender, marital status, rural residence, work status, and whether the respondent spoke English well (all dichotomous variables); respondents’ percentage of life spent in the United States and family income as a proportion of the federal poverty level (continuous variables). We also controlled for immigrants’ country of birth, a variable with the categories “Mexico” (reference), “Guatemala,” “El Salvador,” and “Other,” and highest level of education, a variable with the categories “less than high school” (reference), “high school degree,” “some college,” and “college degree or higher.” We controlled for several health status measures: self-rated health, a categorical variable with categories “poor,” (reference category) “fair,” “good,” “very good,” and “excellent” health, and a count of the number of chronic health conditions a respondent reported, including asthma, diabetes, high blood pressure, and heart disease. Finally, we examined several general health access variables: whether the respondent was insured, whether the respondent had a personal doctor, and respondents’ usual source of healthcare, a variable with the categories “hospital” (reference), “community clinic,” or “no usual source of care beyond the emergency room.”

### 2.4. Statistical analysis

As our preventive care outcome variables are dichotomous, we ran logistic regression models to examine the effects of legal status on preventive care utilization, first as simple models without control variables and subsequently controlling for various demographic and health-related characteristics. We compared the preventive care utilization of naturalized citizens, lawful permanent residents, and temporary status holders to that of undocumented immigrants. We applied sampling weights constructed by CHIS to obtain representative estimates for the California residential population. Analyses were run using Stata software.

## 3. Results

Table 1 presents descriptive statistics for the full 2015–2016 sample (N = 5,513), broken down by legal status (descriptive statistics for our mammogram sample are available in S1 Table in supporting files). Relative to naturalized citizens and LPRs, undocumented immigrants are younger and less likely to be married. They are more likely to be working compared to naturalized citizens but live in households with lower income relative to citizens, LPRs, and temporary status holders. In addition, they report lower levels of education compared to naturalized citizens and temporary status holders and are less likely to speak English well relative to citizens, LPRs, and temporary status holders. Undocumented immigrants in our sample have lived in the United States for a lower percentage of their lives relative to citizens and LPRs, but a higher percentage relative to temporary status holders. Naturalized citizens and temporary status holders are more likely to be from El Salvador relative to undocumented immigrants but are less likely to be from Mexico. Furthermore, undocumented immigrants in our sample differ from naturalized citizens and LPRs but resemble temporary status holders on several health and health access measures. For instance, they report fewer chronic conditions compared to naturalized citizens and LPR respondents, but are less likely to be insured, have a personal doctor, and obtain their usual source of care at a hospital. Instead, when compared to naturalized citizens and LPRs, undocumented immigrants are more likely to report having no usual source of care other than the emergency room.

**Table 1:** Descriptive Statistics.

	Naturalized citizen (40.8%)	Lawful permanent resident (30.2%)	Temporary status (3.4%)	Undocumented (25.6%)
Annual check-up	**77.7%**	**68.7%**	59.3%	52.0%
Flu shot	**47.3%**	**40.4%**	34.4%	29.5%
Mammogram^a^	**84.7%**	**76.8%**	62.3%	63.0%
Female	**55.1%**	50.3%	52.6%	47.3%
Age	**52.9**	**46.5**	39.0	39.2
Income ratio to FPL	**2.4**	**1.8**	**1.5**	1.2
Not working	**37.8%**	31.2%	24.7%	29.8%
Married	**61.9%**	**60.8%**	48.1%	42.2%
Rural	4.8%	**7.7%**	2.1%	4.4%
Education				
Less than HS	**48.2%**	64.6%	**49.9%**	68.4%
HS	21.3%	18.5%	27.6%	20.9%
Some college	**16.1%**	9.1%	6.2%	5.5%
College and above	**14.4%**	7.8%	**16.2%**	5.3%
Country of birth				
Mexico	**73.1%**	81.2%	**55.3%**	82.6%
Guatemala	**4.2%**	5.2%	6.3%	8.7%
El Salvador	**10.5%**	7.7%	**24.5%**	5.7%
Other	**12.2%**	**5.9%**	**14.0%**	3.0%
Percent of life in the US	**63.0%**	**50.9%**	**35.1%**	41.0%
Speaks English well	**52.4%**	**30.5%**	**35.3%**	18.4%
Self-reported health				
Poor	6.7%	5.7%	1.4%	4.7%
Fair	31.8%	32.7%	32.6%	34.0%
Good	31.6%	37.1%	39.8%	37.3%
Very good	**16.6%**	13.1%	11.2%	10.7%
Excellent	13.4%	11.6%	15.0%	13.3%
Chronic conditions	**0.76**	**0.60**	0.47	0.37
Insured	**90.9%**	**84.6%**	61.8%	54.7%
Usual source of care				
Hospital	**52.1%**	**33.0%**	20.8%	14.0%
Community clinic	**31.3%**	38.2%	42.5%	42.5%
None/ ER	**16.6%**	**28.7%**	36.8%	43.5%
Personal doctor	**72.3%**	**58.0%**	33.5%	27.1%

^a^Among women 40 years and older (N = 2,292).

**Boldface** indicates statistically significant (p ≤ 0.05) difference compared with undocumented immigrants.

Our descriptive statistics indicate that undocumented immigrants in our sample are significantly less likely relative to naturalized citizens and LPRs to utilize all the preventive health services we examine, but not relative to temporary status holders. Undocumented immigrants have significantly lower rates of having an annual checkup and receiving a flu vaccine relative to both naturalized citizens and permanent residents. Likewise, undocumented immigrant women aged 40 and older are also less likely to have obtained a mammogram in the past two years relative to their naturalized citizen and permanent resident peers.

Table 2 presents the results from a logistic regression model predicting legal status differences in immigrants’ access to preventive care. Echoing our descriptive findings, we find that relative to undocumented immigrants, naturalized citizens have higher odds of obtaining annual checkups (OR = 3.23, p = 0.000), flu vaccinations (OR = 2.14, p = 0.000) and mammograms (OR = 3.24, p = 0.000). LPRs have similarly higher odds of obtaining these preventive care services compared to undocumented immigrants (checkup OR = 2.03, p = 0.000; flu vaccine OR = 1.62, p = 0.000; mammogram OR = 1.95, p = 0.005). However, we found no statistically significant difference between temporary status holders and undocumented immigrants in their preventive care usage.

**Table 2:** Legal Status Differences in Preventive Care Utilization.

	Annual checkup (simple)	Annual checkup (full)	Flu vaccination (simple)	Flu vaccination (full)	Mammogram (simple)	Mammogram (full)
Legal status						
Naturalized	**3.23** (2.49, 4.17)	**1.45 (1.03, 2.07)**	**2.14** (1.70, 2.70)	1.10 (0.80, 1.51)	**3.24** (2.14, 4.91)	**2.69** (1.57, 4.60)
LPR	**2.03** (1.58, 2.61)	1.23 (0.93, 1.63)	**1.62** (1.27, 2.07)	1.00 (0.76, 1.31)	**1.95** (1.23, 3.10)	**1.68** (1.00, 2.81)
Temporary	1.35 (0.85, 2.15)	1.3 (0.76, 2.23)	1.25 (0.80, 1.96)	1.18 (0.7, 1.86)	0.97 (0.43, 2.19)	1.06 (0.45, 2.49)
None		---	---	---	---	---
Female		**1.36** (1.09, 1.70)		1.21 (0.99, 1.48)		---
Age		1.00 (0.99, 1.00)		**1.01** (1.01, 1.02)		1.01 (0.99, 1.03)
Income ratio to FPL		1.01 (0.96, 1.06)		1.02 (0.97, 1.08)		**1.15** (1.03, 1.28)
Not working		**1.51** (1.19, 1.91)		**1.41** (1.15, 1.73)		1.05 (0.73, 1.52)
Married		0.96 (0.77, 1.20)		0.94 (0.77, 1.14)		1.18 (0.83, 1.69)
Rural		0.93 (0.65, 1.33)		1.30 (0.92, 1.82)		0.56 (0.31, 1.04)
Education						
Less than HS		---		---		---
HS		0.91 (0.70, 1.19)		0.93 (0.72, 1.20)		1.18 (0.76, 1.84)
Some college		1.00 (0.68, 1.48)		**0.66** (0.48, 0.91)		0.83 (0.49, 1.39)
College and above		0.89 (0.57, 1.38)		0.80 (0.57, 1.13)		0.69 (0.35, 1.37)
Country of birth						
Mexico		---		---		---
Guatemala		1.17 (0.77, 1.80)		1.06 (0.74, 1.52)		1.04 (0.56, 1.92)
El Salvador		0.69 (0.45, 1.08)		0.99 (0.70, 1.40)		1.00 (0.56, 1.81)
Other		0.90 (0.62, 1.30)		0.97 (0.69, 1.36)		0.78 (0.42, 1.43)
Percent of life in the US		1.00 (0.99, 1.01)		1.00 (0.99, 1.00)		0.99 (0.98, 1.00)
Speaks English well		1.26 (0.94, 1.69)		0.94 (0.71, 1.23)		0.85 (0.53, 1.37)
Self-reported health						
Poor		---		---		---
Fair		0.78 (0.47, 1.31)		0.68 (0.44, 1.05)		1.56 (0.90, 2.71)
Good		0.73 (0.44, 1.23)		0.78 (0.50, 1.23)		1.17 (0.64, 2.16)
Very good		0.81 (0.45, 1.45)		0.72 (0.43, 1.19)		1.35 (0.64, 2.81)
Excellent		0.74 (0.41, 1.34)		0.81 (0.48, 1.39)		0.81 (0.36, 1.86)
Chronic conditions		**1.48** (1.26, 1.73)		**1.27** (1.12, 1.45)		1.04 (0.84, 1.28)
Insured		**1.83** (1.38, 2.43)		**1.96** (1.49, 2.58)		1.10 (0.70, 1.75)
Usual source of care						
Hospital		---		---		---
Community clinic		1.22 (0.92, 1.62)		1.01 (0.80, 1.27)		1.00 (0.65, 1.54)
None/ ER		**0.69** (0.49, 0.96)		**0.67** (0.48, 0.93)		**0.52** (0.29, 0.93)
Personal doctor		**2.68** (2.04, 3.52)		**1.64** (1.27, 2.12)		1.46 (0.90, 2.34)
Constant		0.71 (0.32, 1.55)		**0.19** (0.09, 0.41)		0.92 (0.23, 3.63)

^a^Among women 40 years and older (N = 2,292).

**Boldface** indicates statistically significant (p ≤ 0.05) difference compared with undocumented immigrants.

While naturalized citizens report significantly higher odds of receiving an annual checkup after adjusting for relevant sociodemographic and health-related controls (OR = 1.45, p = 0.032), annual checkup differences between LPRs and undocumented immigrants and all legal status disparities in flu vaccinations lose statistical significance after controls. Immigrants’ general healthcare access and health status significantly predicted their access to physical exams and flu vaccinations. Latino immigrants who were insured had 1.83 times the odds of obtaining an annual checkup (p = 0.000) and 1.96 times the odds of receiving a flu vaccination (p = 0.000) relative to uninsured immigrants. Those who reported not having a usual source of healthcare other than the emergency room had 0.69 the odds of receiving an annual checkup (p = 0.027) and 0.67 odds of a flu vaccination (p = 0.017) relative to those who reported going to a hospital for their usual source of care. Furthermore, those with a personal doctor reported 2.68 times the odds of receiving an annual checkup (p = 0.000) and 1.64 times the odds of receiving a flu vaccination (p = 0.000) relative to those who did not. Having chronic health conditions also increased immigrants’ likelihoods of receiving preventive care. We found that each reported chronic condition increased immigrants’ odds of receiving an annual exam by 47.5% (p = 0.000) and receiving a flu vaccination by 27.3% (p = 0.000). Finally, immigrants’ sociodemographic characteristics shaped their likelihood of obtaining preventive care. Immigrants who reported being unemployed had 51% higher odds of obtaining an annual checkup (p = 0.001) and 41% higher odds of obtaining a flu vaccine (p = 0.001) relative to those who were employed. Finally, women had higher odds of obtaining an annual checkup (OR = 1.36, p = 0.007) and flu vaccine (OR = 1.21, p = 0.001) relative to men.

Furthermore, we find that legal status differences persist in Latina immigrant women’s mammogram utilization net of socioeconomic, health, and migration controls. Naturalized citizen women have nearly three times the odds (OR = 2.69, p = 0.00) of receiving a mammogram in the past two years relative to undocumented women, while permanent resident women have 1.68 times the odds (p = 0.050). By contrast, we did not find a statistically significant difference in the odds of mammogram utilization between Latina women with temporary status and undocumented Latina women. Women with no usual source of care other than the emergency room reported 0.52 odds of receiving a mammogram relative to women whose usual source of care was in a hospital setting (p = 0.027). Finally, higher family income significantly increased immigrant women’s odds of receiving a mammogram (p = 0.013).

## 4. Discussion

Undocumented immigrants in our sample report lower unadjusted rates of having annual checkups, flu vaccinations, and mammograms relative to permanent residents and naturalized citizens. Naturalized citizens continue to have higher odds of checkups and mammograms, while LPRs remain significantly more likely to have mammograms after controlling for sociodemographic, health status, and health access factors. However, legal status disparities in flu vaccinations attenuate and lose statistical significance after accounting for these elements. Sociodemographic and health factors may explain the relationship between legal status and preventive care access. For instance, we found that immigrants are more likely to obtain checkups and flu vaccines if they are not working. Undocumented immigrants have high levels of employment and may face work-related time constraints that hinder them from seeking preventive services. Furthermore, we found that undocumented immigrants are less likely to be insured and to have a usual source of care and a personal doctor, all factors that may contribute to their reduced preventive care access. Future research should empirically test the role of these factors in mediating legally vulnerable immigrants’ access to preventive care.

Undocumented immigrants are currently excluded from all the provisions of the ACA. While research has shown reductions in healthcare access disparities across various dimensions, including ethnic and socioeconomic status in the years following the ACA, the same patterns may not hold when observing legal status disparities [32,33]. However, it is important to acknowledge that insurance access, ACA policies, and legal status are deeply interconnected rather than wholly distinct spheres. Because insurance eligibility is often contingent on legal status, and the ACA explicitly excluded undocumented immigrants from expanded insurance options, adjusting for insurance status in our models partially adjusts for one of the very mechanisms through which legal status affects care access. Thus, our results should be interpreted with caution, recognizing that insurance coverage may mediate part of the relationship between legal status and preventive care utilization. Previous studies of legal status disparities in immigrants’ preventive healthcare access conducted before the implementation of the ACA found that undocumented immigrants had lower usage rates of preventive services such as mammograms or blood pressure checks [10,20]. We found evidence that legal status disparities in preventive healthcare access persist in the years post ACA implementation. Our results echo previous research finding significant healthcare access disparities following the ACA, which codified the categorical exclusion of undocumented immigrants [19].

Furthermore, we found remaining legal status differences in Latina women’s access to mammogram screenings after accounting for immigrants’ socioeconomic and health access conditions. Undocumented Latinas’ reduced mammography utilization may explain findings from previous research that undocumented Latina patients are diagnosed with more advanced stages of breast cancer relative to documented patients and contribute to breast cancer being the leading cause of cancer death among Latina women [26,28]. Mammogram exams may be viewed as invasive and thus necessitate a strong doctor-patient relationship [27,34]. In addition, past research has found that Latina women may report greater embarrassment with and difficulty understanding mammography procedures compared to non-Hispanic White women [35,36]. We found that undocumented immigrants are significantly less likely to have a personal doctor and to speak English well, factors that may diminish undocumented women’s comfort with receiving a mammogram exam. Paired with barriers such as lack of personal and trust-based relationships with doctors, uninsured status, and poor understanding of mammography procedures, vulnerable immigration status may represent another significant hurdle in obtaining a mammogram exam for Latina women with undocumented and temporary legal status.

Furthermore, studies of mammography utilization among Latina women have noted high levels of cancer fatalism, or the belief that cancer is predetermined and untreatable, as a factor that may hinder mammography rates [34,37]. Legally vulnerable immigrants, who suffer feelings of helplessness and loss of agency due to their precarious legal status [38], may experience elevated perceptions of fatalism that further their lower mammography rates. Nonetheless, we must be cautious when attributing structural and social problems to a concept like fatalism [39]. Additional research should further examine the structural, social, and individual-level factors involved in legally vulnerable women’s lower rates of mammogram testing.

In addition, our findings of pervading legal status disparities in preventive care suggest that beyond socioeconomic and insurance-based constraints, fear of deportation through exposure to hospital settings may prevent undocumented immigrants from seeking preventive health services. Previous research has found that the passage of anti-immigrant legislation and high levels of immigration enforcement led to reduced access to health services among Latino immigrants, particularly for those who are undocumented [40,41]. Even in states with more immigrant-friendly policies, such as California, legally vulnerable immigrants may be deterred from seeking hospital-based care due to fear that their presence in a healthcare setting could increase their risk of detection and deportation. Unlike flu vaccines, which may be distributed at pharmacies or community clinics that do not require government identification or proof of citizenship, mammograms are more often conducted in hospital settings which may appear more unfamiliar and inaccessible to undocumented immigrants. For instance, legally vulnerable immigrants may be deterred from visiting hospitals for fear of discovery and deportation, particularly following several highly publicized cases of US Immigration and Customs Enforcement (ICE) agents arresting legally vulnerable immigrants during hospital appointments [42]. The broader policy climate surrounding immigration enforcement—whether through explicit hospital surveillance policies or the more implicit threat of detection—likely contributes to disparities in healthcare utilization among legally vulnerable populations, even in states where access to public services is relatively more inclusive. Given recent legislative changes mandating hospitals collect immigration status data, legally vulnerable immigrants in California may be deterred from seeking preventive care, mirroring patterns observed in states with restrictive policies.

Finally, our paper is among the first to isolate the preventive health utilization patterns of temporary status holders and identify their low rates of care. We found that while temporary status holders reside in the United States with legal authorization, their preventive care utilization rates resemble those of undocumented immigrants. These results suggest that precarious legal status, rather than outright undocumented status alone, contributes to disparities in preventive care. Most temporary status immigrants in our sample are long-term U.S. residents. Over half have lived in the United States for 15 years or more, and over 70% have lived in the United States for 10 years or more. Long periods under uncertain “liminal” legal statuses may prevent immigrants from utilizing public services that facilitate access to preventive care due to fear of jeopardizing their ability to secure permanent statuses [38]. In 2020, the Trump administration proposed an expansion to the “public charge” rule that would make immigrants who used social services such as Medicaid ineligible for visas and green cards. While this expansion was eventually dismissed, it produced a chilling effect on immigrants’ use of services due to confusion over disqualifying criteria and widespread anti-immigrant rhetoric [43]. Thus, despite temporary status holders being eligible for coverage under the Affordable Care Act and protected from deportation risk, these immigrants have low rates of insurance coverage and preventive care utilization. Future research should further examine medical care utilization among immigrants with temporary legal status in the United States to uncover understudied healthcare vulnerabilities within this population.

Our findings take on heightened urgency in light of recent legislative and policy changes affecting immigrant healthcare access. In 2023 and 2024, states such as Florida and Texas passed legislation mandating that hospitals receiving Medicaid or CHIP funding collect and report patients’ immigration status—policies that may intensify fear and deter undocumented and legally vulnerable immigrants from seeking care, even when services are available. Furthermore, in January 2025, the Trump administration eliminated protections that designated hospitals as “sensitive locations,” shielding them from immigration enforcement actions and raising new uncertainties about whether legally vulnerable immigrants may now face heightened risks when seeking care at hospitals or clinics.

At the federal level, shifting immigration policies and enforcement strategies have signaled a renewed emphasis on border security and interior enforcement, potentially exacerbating the existing “chilling effect” on healthcare utilization among immigrant communities. Relative to care available at pharmacies or pop-up clinics, such as vaccinations, preventive care administered in hospitals, such as mammograms, may see a stronger chilling effect among legally vulnerable immigrants as hospitals become spaces of immigration surveillance. While previous research has documented worsened healthcare access following restrictive immigration policies and increased enforcement efforts, the full impact of these recent measures remains to be seen [18].

Given these developments, our findings also suggest that legal status alone may not fully capture the spectrum of healthcare vulnerabilities faced by immigrant populations. Instead, the broader policy climate—including enforcement measures, surveillance laws, and public discourse—may interact with legal status to shape healthcare-seeking behavior in ways that extend beyond access to insurance or legal eligibility for services. Future research should examine how these evolving policy landscapes affect preventive healthcare utilization and whether they exacerbate existing disparities. Policymakers and healthcare institutions must consider proactive interventions, including sanctuary hospital policies, expanded healthcare navigation programs, and targeted outreach efforts, to mitigate the chilling effects of restrictive immigration measures. Given the critical role of preventive care in population health, ensuring equitable access for legally vulnerable immigrants remains an urgent public health and ethical imperative.

### 4.1. Limitations

Our study is limited to immigrants in California, a state with some of the most immigrant-friendly policies in the nation [44]. Starting in January 2024, California became the first state to expand access to Medicaid (known as Medi-Cal in California) to all low-income residents, regardless of legal status. Our findings that legal status disparities in preventive care exist in California may therefore be magnified in states with more restrictive policies towards immigrants. For instance, while our California data did not show statistically significant legal status disparities in flu vaccinations or annual checkup rates between permanent residents and undocumented immigrants after sociodemographic controls, there may be remaining disparities in states like Florida and Texas that mandate hospitals to collect patients’ immigration status.

Our analyses, based on 2015–2016 data, captures a key transitional period following the ACA’s initial implementation but before recent expansions to Medi-Cal in California that would limit the generalizability of our findings to states without expanded public insurance programs for undocumented residents. These findings provide critical historical insight into understanding how legal status disparities persisted even during major policy changes intended to broaden healthcare access. However, our analyses do not capture policy contexts post-2016, particularly the environment of heightened deportation fears and diminished public programs for legally vulnerable immigrants following the election of Donald Trump. For instance, in May 2025, Governor Gavin Newsom proposed freezing Medi-Cal eligibility among undocumented immigrants in 2026, highlighting the continued vulnerability of undocumented immigrants even in California. Future studies should examine legal status patterns in preventive care access in other states, trends in immigrants’ preventive care uptake in more recent years, whether the expansion of Medi-Cal has mitigated legal status disparities in preventive care usage, and the implications of ending Medi-Cal eligibility for undocumented immigrants.

## 5. Conclusion

Despite the significance of preventive care for maintaining and improving health, immigrants face barriers to preventive care access in the United States. Due in part to limited data, we have little empirical knowledge of how immigrants’ access to preventive care may differ by their legal status. This paper examines legal status disparities in the utilization of preventive care among Latino immigrants in California. We found that undocumented immigrants report lower unadjusted rates of obtaining annual checkups, flu vaccinations, and mammogram screenings relative to citizen and permanent resident immigrants, but similar rates relative to temporary status immigrants. Legal status disparities in obtaining flu vaccinations lost statistical significance after adjusting for sociodemographic and health access controls, particularly insurance status, access to hospitals for usual source of care, and having a personal doctor. Nonetheless, differences between citizens and undocumented immigrants in obtaining annual checkups and legal status disparities in mammogram screenings persisted after the inclusion of health access and demographic controls. However, migrants with temporary legal status report comparable rates of preventive care utilization relative to undocumented immigrants, despite their protected legal status and their eligibility for health programs that exclude the undocumented. Our findings demonstrate the need to address access to and utilization of preventive care for legally vulnerable immigrant populations, including immigrants with temporary legal status.

## Supporting information

S1 Fig Preventive care utilization by year. (DOCX)

S1 Table Descriptive statistics for mammogram sample (N = 2,292). (DOCX)
