# Supplementary figures and images for: Legal status disparities in preventive care usage among Latino immigrants in California: A cross-sectional analysis
Source: PLOS Glob Public Health. 2025 Jul 1;5(7):e0004855. doi: 10.1371/journal.pgph.0004855 (PMC12212483; doi:10.1371/journal.pgph.0004855)

S1 Fig


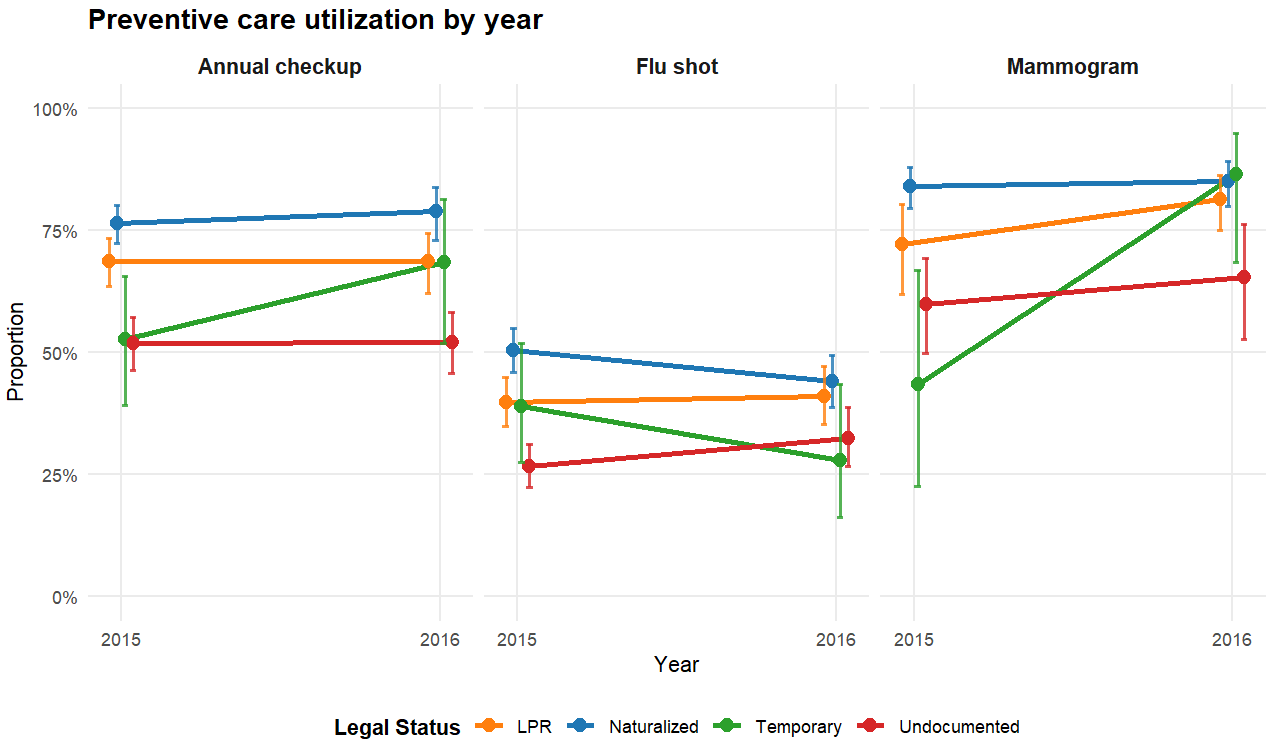

Supplement: S1 Fig — (DOCX) [file pgph.0004855.s001.docx]
