# Supplementary material for: Legal status disparities in preventive care usage among Latino immigrants in California: A cross-sectional analysis
Source: PLOS Glob Public Health. 2025 Jul 1;5(7):e0004855. doi: 10.1371/journal.pgph.0004855 (PMC12212483; doi:10.1371/journal.pgph.0004855)
Supplement: S1 Table — (DOCX) [file pgph.0004855.s002.docx]

S1 Table: Descriptive statistics for mammogram sample (N = 2,292)

|  | Naturalized citizen (47.9%) | Lawful permanent resident (29.0%) | Temporary status (2.3%) | Undocumented (20.1%) |
| --- | --- | --- | --- | --- |
| Mammogram | **84.7%** | **76.8%** | 62.3% | 63.0% |
| Age | **58.7** | **52.2** | 47.3 | 47.5 |
| Income ratio to FPL | **2.1** | **1.5** | 1.3 | 1.1 |
| Not working | 52.1% | 41.6% | 42.8% | 51.6% |
| Married | **56.8%** | **66.9%** | 61.0% | 45.0% |
| Rural | 4.4% | **8.2%** | 2.8% | 2.6% |
| Education |  |  |  |  |
| Less than HS | **53.5%** | 79.0% | 68.3% | 72.1% |
| HS | 20.2% | **11.2%** | 14.5% | 20.3% |
| Some college | **15.1%** | 5.9% | 6.2% | 3.6% |
| College and above | **11.1%** | 3.3% | 11.0% | 3.9% |
| Country of birth |  |  |  |  |
| Mexico | **70.7%** | 83.7% | **65.6%** | 88.3% |
| Guatemala | 3.8% | 3.4% | 1.1% | 3.2% |
| El Salvador | 11.0% | 7.6% | 19.0% | 5.2% |
| Other | **14.5%** | 5.4% | 14.3% | 3.3% |
| Percent of life in the US | **60.3%** | **48.4%** | 42.1% | 39.5% |
| Speaks English well | **42.6%** | 18.5% | 9.0% | 12.0% |
| Self-reported health |  |  |  |  |
| Poor | 8.5% | 7.8% | 6.8% | 7.7% |
| Fair | 38.0% | 42.9% | 47.2% | 44.3% |
| Good | 28.2% | 35.0% | 35.5% | 33.2% |
| Very good | **14.9%** | 6.7% | 4.1% | 4.4% |
| Excellent | 10.5% | 7.6% | 6.4% | 10.4% |
| Chronic conditions | **0.86** | **0.75** | 0.69 | 0.52 |
| Insured | **91.1%** | **85.9%** | 49.8% | 61.9% |
| Usual source of care |  |  |  |  |
| Hospital | **59.0%** | **32.6%** | 22.5% | 12.7% |
| Community clinic | **28.7%** | 45.3% | 38.5% | 55.7% |
| None/ ER | **12.3%** | 22.1% | 39.0% | 31.6% |
| Personal doctor | **79.7%** | **70.2%** | 34.6% | 42.3% |
| **Boldface** indicates statistically significant (p ≤ 0.05) difference compared with undocumented immigrants | | | |  |
